# Comparative study on archaeal diversity in the sediments of two urban landscape water bodies

**DOI:** 10.1371/journal.pone.0229097

**Published:** 2020-02-18

**Authors:** Yuxin Wang, Chaonan Wang

**Affiliations:** College of Water Resources & Civil Engineering, China Agricultural University, Haidian, Beijing, China; Free University of Bozen/Bolzano, ITALY

## Abstract

Urban lake sediment plays a very important role in waterbody ecosystems. It is the basis of nutrient cycling and material exchange between microorganisms and lake ecosystems. In this study, Illumina high-throughput sequencing technology was employed to detect the structure and species richness of Archaea in anoxic sediments of urban waterbodies (Aohai Lake and Kunming Lake) in Beijing, and the environmental factors (pH level, organic matter, available nitrogen and total nitrogen) affecting the structure and succession of archaeal communities were also investigated. The results showed that there were 13 classified archaeal phyla, and the most frequent archaeal species in the lakes were *Bathyarchaeota (MCG)*, *Euryarchaeota*, *Thaumarchaeota*, *Aenigmarchaeota*, *Hadesarchaea*, *Lokiarchaeota*, and *Parvarchaeota*. The top ten most abundant genera in the two lakes were significantly associated with at least one environmental factor. The results of this study enrich the understanding of microbial diversity in urban eutrophic lake sediments in northern China.

## Introduction

Lake eutrophication is a major environmental problem in the field of water resources worldwide [1], and sediment is an important source of lake nutrients [2]. Geochemical processes such as the carbon cycle, nitrogen cycle and phosphorus cycle in sediments affect or determine the species and diversity of microbial communities [3]. Archaea play an irreplaceable role in the global biogeochemical cycle because their characteristics are different from those of bacteria and eukaryotes [4, 5]. The cell wall of Archaea does not contain peptidoglycan, the cytoskeleton is composed of protein or pseudopeptidoglycan, and the cell membrane is composed of glycerol molecules and branched hydrocarbons linked by ether bonds [6].

Traditionally, it is generally believed that Archaea live in extreme environments, such as hot springs and anaerobic environments. With the rapid development of molecular methods independent of culture, increasing research has proven that Archaea exist in various environments on Earth. Archaea are more widely distributed and metabolically diverse than previously thought. Initially, there were only two phyla in Archaea; to date, this number has increased to 28 phyla [6], including *Euryarchaeota*, *Crenarchaeota*, *Nanoarchaeota*, *Thaumarchaeota*, *Korarchaeota*, *Parvarchaeota*, *Lokiarchaeota*, *Thorarchaeota*, *Woesearchaeota*, *Verstraetearchaeota*, *Bathyarchaeota and Odinarchaeota*, etc. Taking *Bathyarchaeota* as an example, with the development of macrogenomics, a large number of *Bathyarchaeota* have been found distributed in freshwater lakes, estuarine sediments, salt marshes, groundwater and other habitats, which proves the wide distribution characteristics of this microorganism.

*Euryarchaeota* contain methanogens, extreme halophilic bacteria and thermophilic acidophiles, are widely distributed and have diverse physiological characteristics [7, 8]. *Crenarchaeota* [9] are mainly thermophilic bacteria, representing the most primitive archaeological groups located in the root of phylogenetic trees, such as *Thermococcus* and *Pyrodictium*. The first ammonia-oxidizing archaea (AOA) strain was isolated and cultured from seawater of the Seattle Aquarium in 2005 [10], which confirmed that Archaea could also perform ammonia oxidation. Subsequently, a series of molecular ecology studies showed that ammonia-oxidizing archaea (AOA) were widely distributed in a variety of environments, including oceans, sediments, freshwater systems and soils. The abundances of AOA are generally much higher than those of ammonia-oxidizing bacteria (AOB), indicating that AOA might play an important role in nitrification.

A large number of studies have shown that up to 99% of microorganisms in nature cannot be cultured, and it is difficult to obtain all the information of microbial community diversity by traditional pure culture and isolation methods [11]. High-throughput technology can be used to investigate the diversity of microbial communities at the genetic level and can effectively overcome the limitations of traditional microbial culture techniques. Through 16S rRNA gene sequencing technology, the response of the microbial community structure composition to the environment and the adaptive mechanism and metabolic pathway of lake microorganisms under extreme environmental conditions can be explored.

## Materials and methods

### Study area and sample collection

Samples for this study were collected from two artificial lakes in Beijing: the Kunming Lake and the Aohai Lake. Kunming Lake is an important scenic water area in Beijing. It is located in the Summer Palace in the west of Beijing. Its water surface area is 194.0 hm^2^. It is the largest lake in Beijing urban area, accounting for 39% of the total Lake area in the city. However, the surface of Kunming Lake is narrow, its water depth is shallow, and its average water depth is about 1.5m. Aohai Lake is the name of the main lake (built in 2007) in Beijing Olympic Forest Park which covers an area of 28.7 hm^2^. There is no natural water system flowing in Aohai Lake except for rainwater supply in rainy season. It mainly uses the reclaimed water from Qinghe sewage treatment plant located on the west side of the park as the supply water source[12]. The water environment quality of public water body has a great impact on the health of urban residents. At present, the systematic study of microbial communities in urban eutrophic water is still limited. It is of great significance to study the community structure of *Archaea* in water environment and sediment for eutrophication biological monitoring and ecological restoration of urban water bodies.

The sediment samples (20–25 cm below the surface of the water) from 6 sites were collected from Kunming Lake and Aohai Lake in March of 2018 (Table 1). There were three sampling points in the Kunming Lake, which were KU (situated at the inlet of Kunming Lake), KM (situated at the lake area of Kunming Lake) and KL (situated at the outlet of Kunming Lake). Similarly, there were also three sampling points in the Aohai Lake, which were AU (situated at the inlet of Aohai Lake), AM (situated at the lake area of Aohai Lake) and AL (situated at the outlet of Aohai Lake). On the sampling day, the water temperature of the sampling points in the two lakes is 11 °C. There were triplicate samples that were collected from each sampling point. A total of 18 samples were collected under aseptic sealing conditions, placed in a container on ice and transported to the laboratory within 1 h. We divided the samples into two halves. One half was processed immediately for measurements of sediment physicochemical parameters; the other half was stored in sterile polypropylene tubes at -80°C for molecular analysis.

**Table 1:** Physicochemical characteristics of sediment samples from two lakes in this study.

Sites	Longitude and Latitude	pH	OM (mg/g)	TN (mg/g)	AN (mg/g)	AP (μg/g)
KU	116°15′41″E,39°59′39″N	7.47	7.69	61.0	0.096	1.81
KM	116°15′45″E,39°59′3″N	8.11	13.42	86.6	0.133	1.43
KL	116°16′26″E,39°58′50″N	8.07	17.29	82.8	0.119	2.39
AU	116°23′31″E,40°0′42″N	8.62	25.31	67.0	0.087	3.35
AM	116°22′55″E,40°0′48″N	8.25	10.27	59.1	0.059	2.20
AL	116°22′50″E,40°1′4″N	7.87	26.60	114.2	0.121	3.15

### Physiochemical analysis

Sediment pH was determined by a pH meter (solid to water ratio was 1:2.5). Organic matter (OM) was determined by the dichromate oxidation process. Available nitrogen (AN) was determined using the alkali-hydrolysed diffusing method. Total nitrogen (TN) was analysed by the Semi-Micro Kjeldahl Method. Available phosphorus (AP) was extracted with NH_4_F-HCl solution and then determined by an ultraviolet visible spectrophotometer.

### DNA extraction and sequencing

Total DNA was extracted from 500 mg of sediment sample using the Qiagen QIAamp Fast DNA Stool Mini Kit according to the manufacturer’s protocols. The final DNA concentration and purification were determined by a NanoDrop 2000 UV-vis spectrophotometer and run on a 1.0% agarose gel before being used for sequencing. The extracted DNA was amplified using the primers 524F (5’-TGYCAGCCGCCGCGGTAA-3’) and Arch958R (5’-YCCGGCGTTGAVTCCAATT-3’) [13], which target conserved sequences found in archaea. All PCR amplicons were isolated from 2% agarose gels and purified with a DNA gel extraction kit (Axygen Biosciences, Union City, CA, USA). The DNA concentration of each PCR product was determined using a QuantiFluor^™^-ST fluorescent quantitative system (Promega, USA) before sequencing. The obtained raw sequences were analysed using Trimmomatic and merged by FLASH [14]. Then, the remaining sequences were clustered into operational taxonomic units (OTUs) at 3% divergence implemented using UPARSE (version 7.1 http://drive5.com/uparse/), and chimeric sequences were identified and removed using UCHIME [15].

All PCR products were sequenced using the Illumina MiSeq platform (Illumina, San Diego, USA) by Shanghai Majorbio Bio-pharm Technology Co., Ltd., China. The raw reads were deposited into the NCBI Sequence Read Archive database.

### Statistical analyses

Alpha-diversity analyses, including community richness parameters (Chao1, ACE), community diversity parameters (Shannon, Simpson), and a sequencing depth index (Good’s coverage), were calculated by Mothur software [16]. Rank-abundance curves and Archaeal taxonomic distributions of sample communities were visualized by the R package. A Venn diagram was implemented by the R package to show unique and shared OTUs. Phylogenetic trees were calculated using the approximate maximum likelihood method with 500 bootstrap replicates in FastTree (version 2.1.3 http://www.microbesonline.org/fasttree/). To investigate the relationships between the sediment archaeal community and the environmental variables, redundancy analysis (RDA) with Bray-Curtis distance was carried out by the RDA analysis package of R language. Spearman’s correlation analysis was performed among the top 10 genera (based on the rank of relative abundance) and environmental variables. Then, a heatmap was produced with the “heatmap” package in R.

## Results

### The diversity of the archaeal community

**Fig 1:**
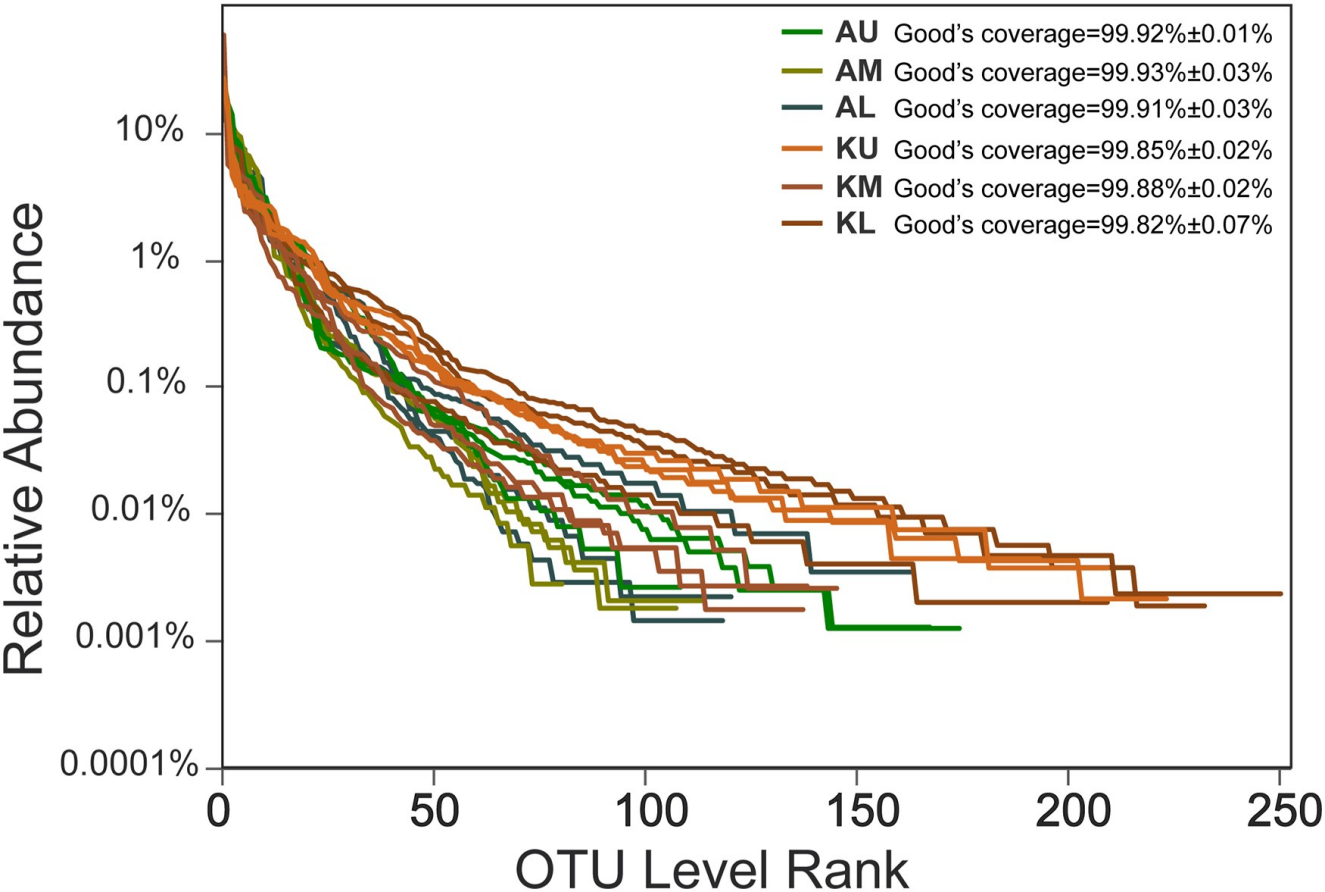
Rank-abundance curves for the archaeal community in the sediments of lakes.

A total of 1,065,716 high-quality reads were obtained from the 18 samples after quality filtering, ranging from 30,954 to 98,117 reads per sample. Sample coverage was estimated at 99% for each sample, which indicated that the sequencing depth was high and that the data were reliable (Fig 1). A total of 415 OTUs were observed for all samples, from 81 to 251 OTUs for each sample.

Rank-abundance curve is a chart used by ecologists to display relative species abundance that can be used to visualize species abundance, a component of biodiversity. The width of the curve reflected the abundance of the species. The smoother the curve, the more uniform is the species distribution [17]. As shown in Fig 1, the width of curve in sampling points KL and KU was larger than that in AU, AM, AL and KM, namely indicated the species more abundant in the sampling points KL and KU. And furthermore, it also appeared more uniform of the species distribution in the sampling points KL and KU. Compared with the other sampling points, the abundance and uniformity of species in the sampling point AM were lower.

**Fig 2:**
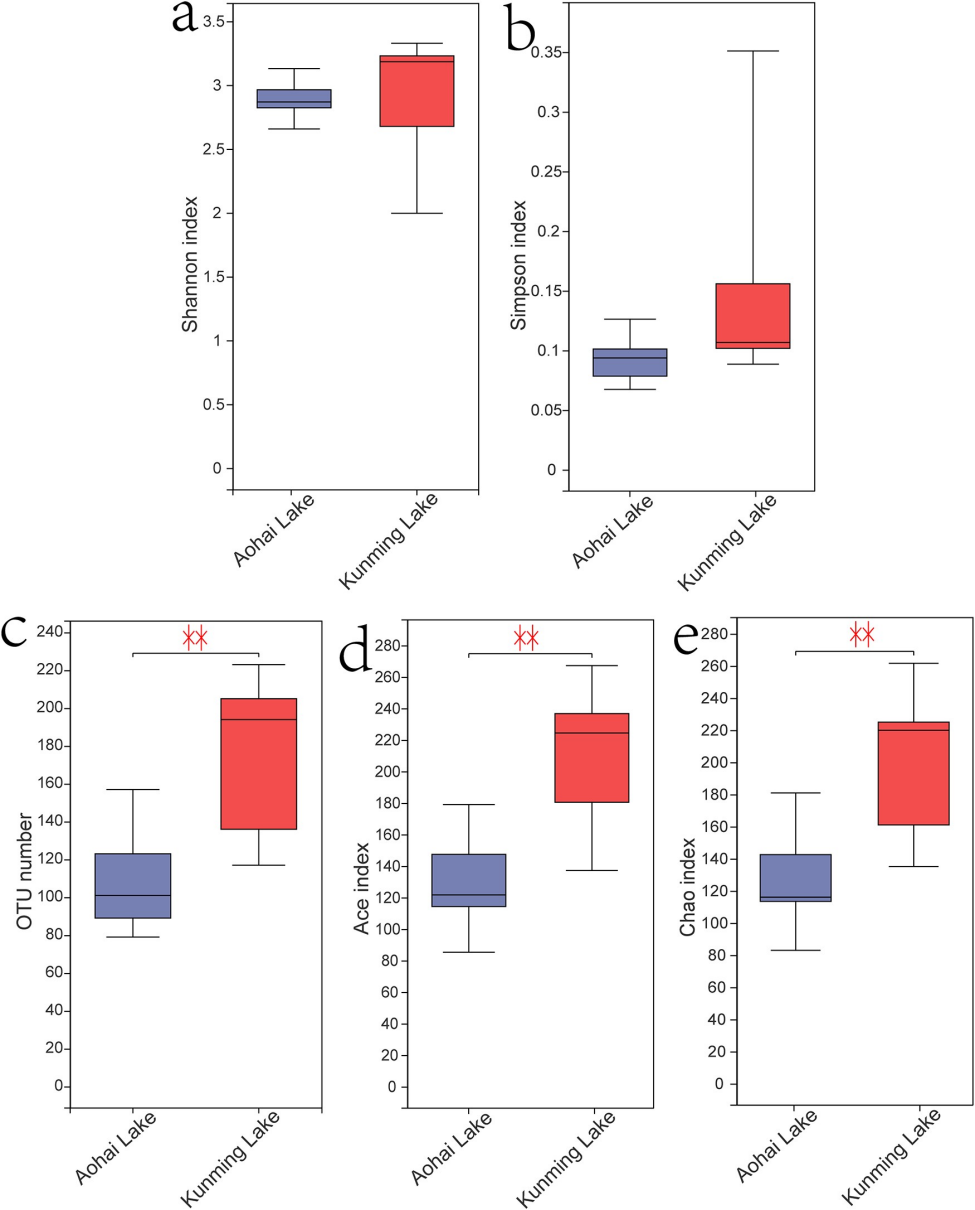
Microbial OTU number and diversity index in different lakes. The number of OTUs, the richness estimators Chao and Ace, and the diversity estimators Shannon and Simpson were calculated at a 3% distance. *P < 0.05, **P < 0.01. OTU, operational taxonomic unit.

In this study, Shannon, Simpson, Ace and Chao1 indexes were calculated to estimate and compare the sediment archaeal richness and diversity in different water sources and locations (Fig 2 and Table 2). No significant changes in sediment archaeal diversity were observed among the two lakes (p > 0.05). However, compared with the Kunming Lake (p < 0.01), a significant decreasing trend in species richness at the Aohai Lake was observed.

**Table 2:** Richness and diversity indexes relative to each part of the lake.

**Richness and diversity indexes relative to each part of the Aohai Lake**						
Estimators	AU	AM	AL	P-value (AM-AU)	P-value (AL-AU)	P-value (AL-AM)
Sobs	120.33±17.16	89.333±11.06	115.67±36.295	0.081	0.663	0.268
Shannon	2.888±0.230	2.824±0.15	2.97±0.151	1	1	0.383
Simpson	0.098±0.029	0.089±0.014	0.087±0.018	1	0.663	1
Ace	135.45±12.976	115.31±39.303	138.33±35.39	0.663	1	0.383
Chao	135.17±18.836	105.22±24.607	136.77±38.224	0.19	1	0.383
**Richness and diversity indexes relative to each part of the Kunming Lake**						
Estimators	KL	KM	KU	P-value (KL-KM)	P-value (KM-KU)	P-value (KL-KU)
Sobs	206±19.31	126.67±9.5	201.33±6.35	0.081	0.077	0.658
Shannon	3.115±0.309	2.448±0.392	3.204±0.023	0.081	0.081	0.663
Simpson	0.113±0.016	0.232±0.105	0.095±0.008	0.081	0.081	0.190
Ace	245.87±21.41	159.86±21.69	227.97±7.76	0.081	0.081	0.383
Chao	243.68±21.49	149.73±13.29	221.83±3.415	0.081	0.081	0.383

Data represent the mean ± standard deviation (S.D.) Statistical analyses were performed with the Wilcoxon rank sum test between the two groups. The number of OTUs, richness estimators Chao and Ace, and diversity estimators Shannon and Simpson were calculated at a 3% distance.

*P < 0.05,

**P < 0.01.

OTU, operational taxonomic unit.

**Fig 3:**
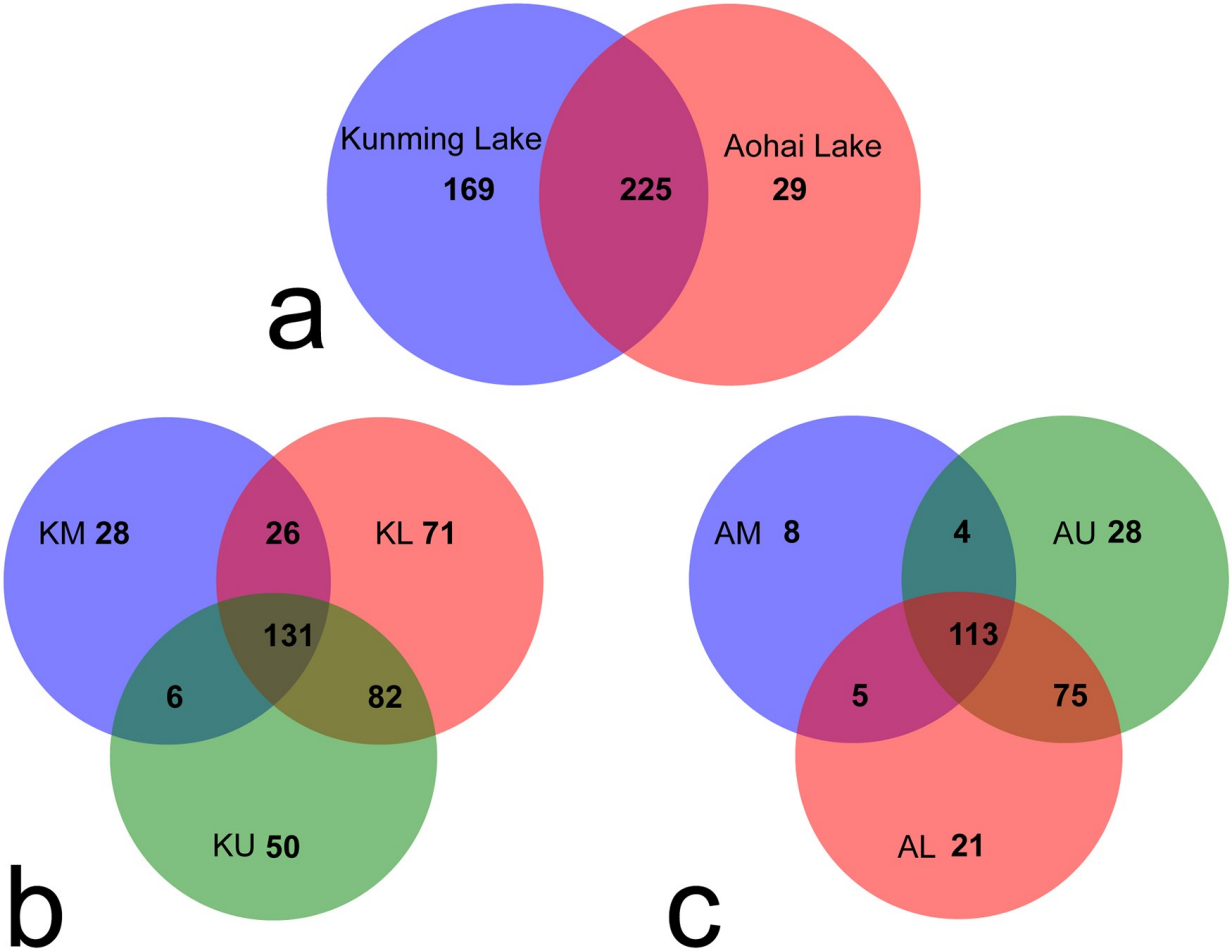
Unique and shared OTUs presented in the Kunming Lake and the Aohai Lake. The overlapping portion indicates OTUs shared in different sample groups or sampling points; the nonoverlapping portion indicates OTUs unique to one sample group or sampling point, and the figure on the graph indicates the corresponding number of OTUs.

Next, we examined the shared and unique archaeal taxa between the microbiotas from the two groups (Kunming Lake vs. Aohai Lake) based on our sequencing data. A total of 225 shared OTUs were identified from the 18 samples, as shown in Fig 3a. This result indicated that the OTU categories representing the Kunming Lake (394) were higher than those of the Aohai Lake (254). As shown in Fig 3b, there were 131 identical OTU categories in 9 samplings, accounting for 33.3% of the total OTUs in the group of the Kunming Lake. It was obvious that there were 310 OTU categories in the KL (sample point), accounting for 78.9% of the total OTUs in the Kunming Lake. As shown in Fig 3c, we observed 113 identical OTU categories that accounted for 44.5% of the total number of OTUs in the Aohai Lake samples. The AU (sample point) was rich in unique OTU numbers, accounting for 86.6% of the total OTUs in the Aohai Lake group.

### The overall structure and composition of the archaeal community

**Fig 4:**
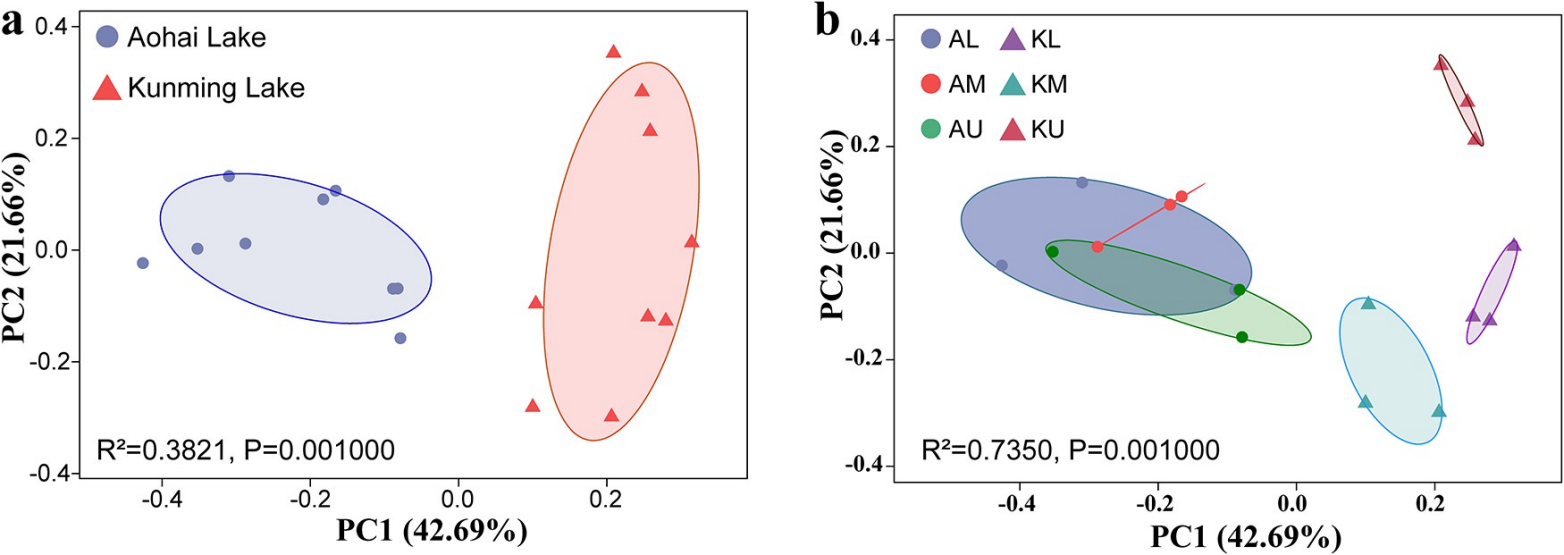
Archaea are separable by the type of water bodies at the OTU level. Statistical support for the PCoA clustering is provided by the analysis of similarity (ANOSIM). The corresponding shaded area in the group shows the 95% confidence interval with 1000 permutations.

Unconstrained principal coordinate analyses (PCoAs) of Bray-Curtis distance were performed to investigate patterns of separation between archaeal communities (Fig 4). Based on the PCoAs, the two communities were separated across the first principal coordinate, indicating that the largest source of variation in archaeal communities of sediments is the type of water body (Fig 4a). To statistically support the visual clustering of the archaeal communities in the above PCoAs, the communities of the two lakes were examined using ANOSIM (an analogue of univariate ANOVA) with the Spearman rank correlation method. The two lakes rendered archaeal microbiota significantly dissimilar from each other (R^2^ = 0.3821, P = 0.001). When the distribution of sampling point in the lake was taken as the grouping factor (Fig 4b), the Aohai Lake samples gathered, but the Kunming Lake samples separated from each other. ANOSIM with the Bray-Curtis distance algorithm (R^2^ = 0.735, P = 0.001) indicated that the distribution of sampling point in the lake as the grouping factor could lead to more significant differences in the calculation results.

**Fig 5:**
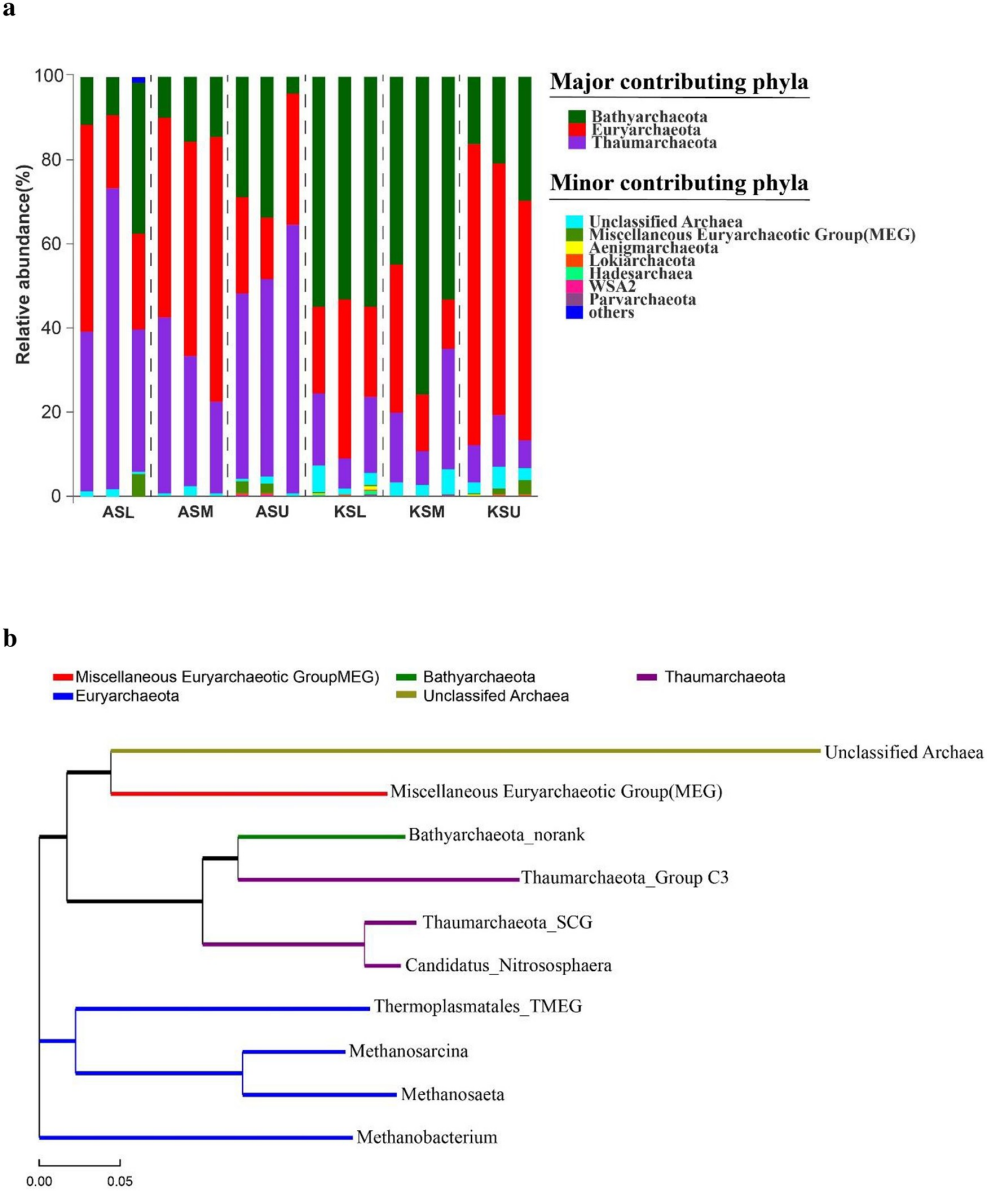
(a) Community bar-plot analysis shows the relative abundance of archaea in each sample at the phylum level. (b) Phylogenetic analysis of the top 10 most abundant genera. Maximum-likelihood tree constructed on the basis of 16S rRNA gene sequences. Bootstrap values were obtained from a search with 500 replicates. Each colour represents a different phylum.

The archaeal community composition varied among the different sampling sites. A total of 13 archaeal phyla were identified across the entire sampling points. In the Kunming Lake groups, *Bathyarchaeota* (25.00% to 78.56%) became the most abundant phylum, followed by *Euryarchaeota* (10.68% to 63.97%) and *Thaumarchaeota* (5.82% to 25.58%). However, *Thaumarchaeota* (19.52% to 71.90%) was the most dominant phylum in the Aohai Lake groups, and the second largest phylum was *Euryarchaeota* (13.02% to 56.43%). *Bathyarchaeota* (8.66% to 40.74%) was the third largest phylum (Fig 5a). Other phyla that were consistently detected included the *Miscellaneous Euryarchaeotic Group* (MEG), *Aenigmarchaeota*, *Lokiarchaeota*, *Hadesarchaea*, WSA2 and *Parvarchaeota*. Rare phyla, defined as those with a relative abundance of less than 0.1%, were clustered together in the “other” category.

At the genus level, detected OTUs were distributed among 56 different archaeal genera in total. In the Kunming Lake samples, 53 genera were detected, and in the Aohai Lake samples, 51 genera were detected. Table 3 shows the top ten most abundant genera and their percentages in both the Kunming Lake and the Aohai Lake. The percentages of the total community covered by the top ten genera ranged from 89.7% (KU) to 98.7% (AU). Then, the evolutionary relationship of the top ten most abundant genera was analysed. The phylogenetic tree (Fig 5b) indicated that the top ten most abundant genera belong to five phyla. *Methanobacterium*, *Methanosarcina*, *Methanosaeta*, and *Thermoplasmatales*_TMEG belong to *Euryarchaeota*, and *Thaumarchaeota*_SCG, *Thaumarchaeota*_Group C3 and *Candidatus Nitrososphaera* belong to *Thaumarchaeota*.

**Table 3:** The top 10 most abundant genera in all of the samples from the two lakes.

Genus	Percentage (%)					
	AU	AM	AL	KU	KM	KL
*Bathyarchaeota_norank*	33.9	22.9	14.9	28.9	60.7	59.1
*Thaumarchaeota_SCG*	35.5	25.2	43	0.7	8.8	2.7
*Methanobacterium*	11.7	32.8	18.1	13.2	11.3	11.9
*Methanosarcina*	1.7	7.3	5.8	24.9	5	6.6
*Thaumarchaeota_Group C3*	5.5	0.7	1.8	7	5.6	8.3
*Methanosaeta*	3.8	6.7	2.6	8.5	0.1	2.6
*Candidatus Nitrososphaera*	2.8	0.9	8.6	0.1	1.1	1.2
*Unclassified Archaea*	1	1	1.3	3	3.4	3
*Thermoplasmatales_TMEG*	1	1.8	0.8	2.2	1.8	1.2
*Miscellaneous Euryarchaeotic Group (MEG)*	1.8	0.1	1.1	1.2	0.1	0.1
**Total**	**98.7**	**99.4**	**98**	**89.7**	**97.9**	**96.7**

### Correlation of environmental variables with archaeal richness

Table 1 lists the environmental parameters, including water temperature, pH, organic matter (OM), total nitrogen (TN), available nitrogen (AN), and available phosphorus (AP). Environmental parameters changed largely among the sediment samples from the 6 sites.

**Fig 6:**
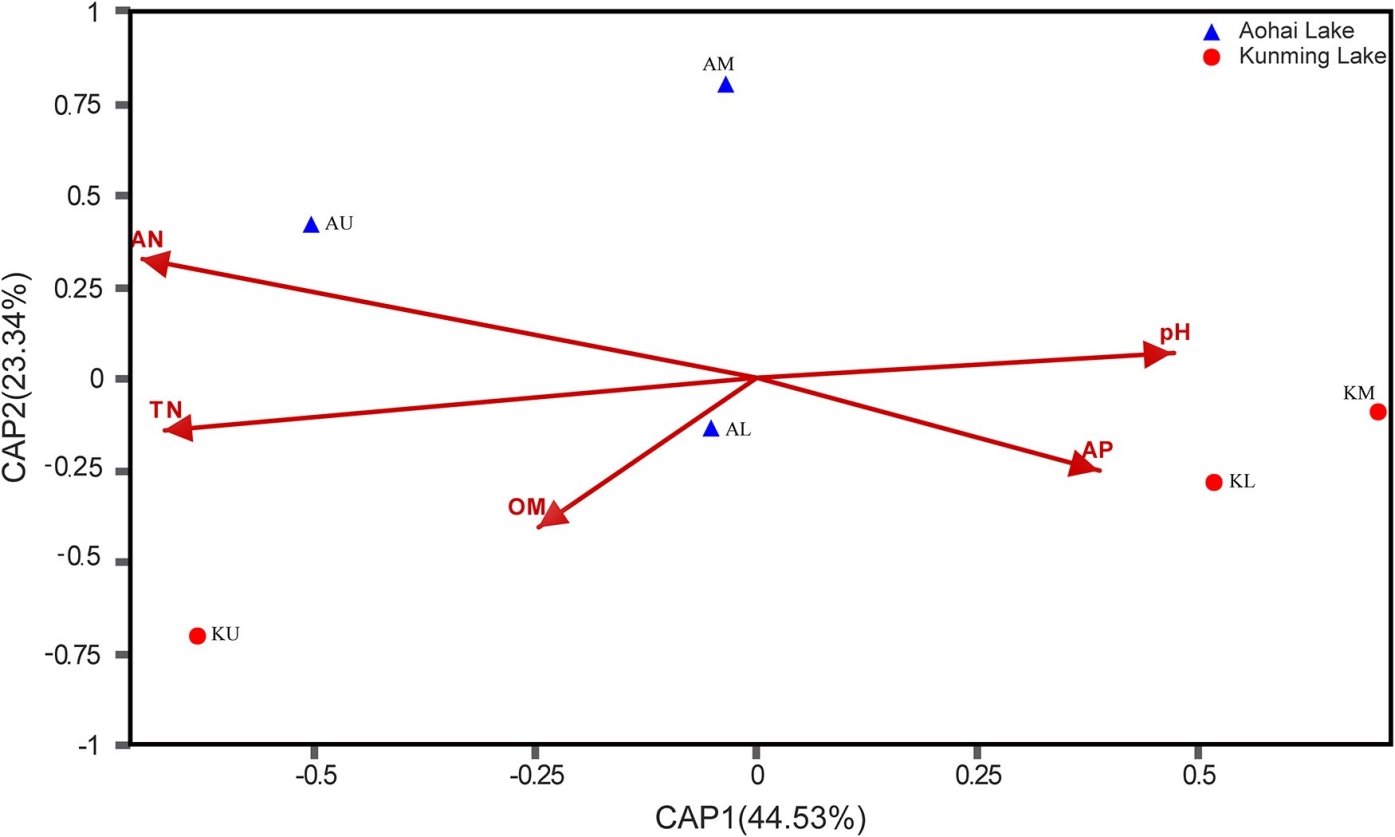
The relationship of archaeal community composition with the major environmental factors at the phylum level. The environmental factors and different groups are indicated by red arrows and different shapes, respectively.

Redundancy analysis (RDA) was used to determine the relationship between the samples and environmental variables (Fig 6). The first RDA dimension explained 44.53% of the variation in archaeal communities, and the second explained 23.34%. The environmental variable that contributed significantly to the archaeal community-environment relationship was AN (p = 0.015).

**Fig 7:**
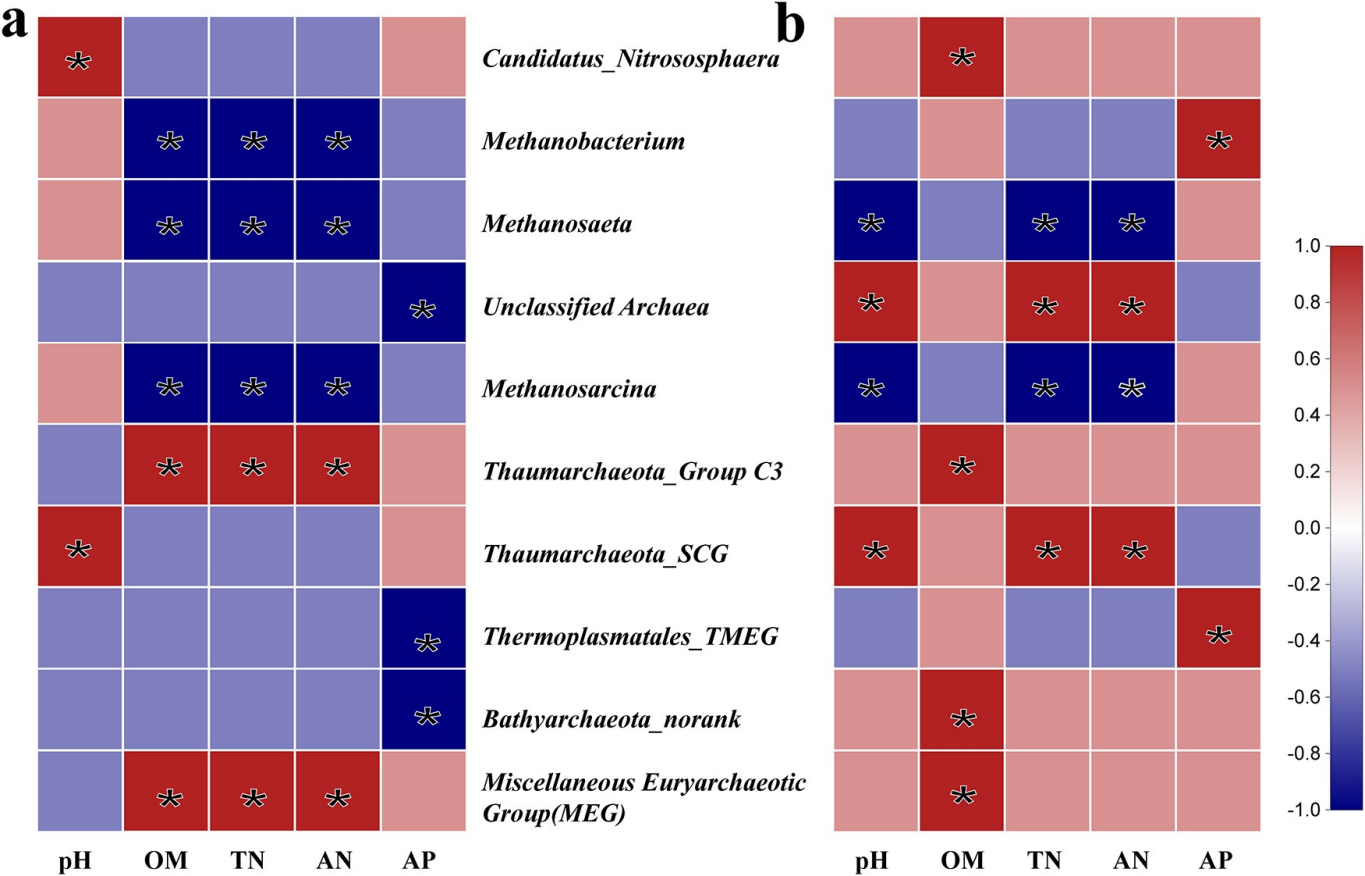
Correlation heat map of the top ten genera and sediment properties. The X and Y axes are environmental factors and genera, respectively. R values are shown in different colours; the right side of the legend indicates the colour range of the different R values. a: Aohai Lake; b: Kunming Lake. Significant values are shown as *P<0.001.

Generally, most *Archaea* are restricted to specific niches, mainly due to different environmental elements [18–20]. The correlation heat map showed that the parameters of sediments, such as pH level, organic matter (OM), available nitrogen (AN), and total nitrogen (TN), had a significant effect on a considerable number of high-abundance species and may be the main factors shaping the microbial community structure of archaea (Figs 6 and 7). The top ten most abundant genera in the two lakes were significantly associated with at least one environmental factor. Both *Methanosaeta* and *Methanosarcina* demonstrated a significant negative correlation with TN and AN. *Thaumarchaeota*_Group C3 and *Miscellaneous Euryarchaeotic Group* (MEG) showed a significant positive correlation with OM, while *Thaumarchaeota*_SCG was positively correlated with pH.

## Discussion

Aquatic sediments are important sites for elements cycling and energy metabolism, and information about the composition of the microbial community structure in sediments is very important for understanding the metabolic processes of aquatic ecosystems.

Firstly, in this research, at the level of genera, *Bathyarchaeota_norank* was quite abundant in both Aohai and Kunming Lake. At three sampling points of Aohai Lake, namely AU, AM and AL, the abundances of *Bathyarchaeota_norank* were 33.9%, 22.9% and 14.9% respectively. In the other three sampling points of Kunming Lake, namely KU, KM and KL, the abundances of *Bathyarchaeota_norank* were 28.9%, 60.7% and 59.1%, respectively.

*Bathyarchaeota* has a variety of physiological and biochemical functions, which can degrade organic matter such as protein, polycarbonate and fatty acid, participate in methane metabolism cycle, produce acetic acid, dissimilate and reduce nitrite and sulfate, which is probably one of the important driving forces of carbon cycle in the earth [21]. However, the complete metabolic pathway, the distribution rule of each subgroup of *Bathyarchaeota*, and their influence on the material circulation and energy circulation of water ecological environment need to be further explored.

Secondly, *Thaumarchaeota_SCG* possessed higher abundance in the lake of Aohai. In the three sampling points of AU, AM and AL, the abundances of *Thaumarchaeota_SCG* were 35.5%, 25.2% and 43% respectively. Compared with Aohai Lake, the abundance of *Thaumarchaeota_SCG* in Kunming Lake is lower. The abundances of *Thaumarchaeota_SCG* in the other three sampling points KU, KM and KL were 0.7%, 8.8% and 2.7%, respectively. *Thaumarchaeota* [22–24] can oxidize ammonia to nitrite and the ammonia oxidizing process is a rate-limiting step of the nitrification process [25, 26]. Thus, our results suggest that the phylum *Thaumarchaeota* may play a more critical role in nitrogen cycling in the sediment of these two lakes.

Moreover, *Candidatus Nitrososphaera* also belongs to ammoxidation *Archaea* (AOA), which is related to the characteristics of adapting to high nitrogen environment [27]. In the sampling point AL of Aohai Lake, the abundances of *Candidatus Nitrososphaera* reached 8.6%. At the same time, in the sampling point KL of Kunming Lake, the abundances of *Candidatus Nitrososphaera* reached 1.2%.

In conclusion, a highly diverse archaeal community was found in the sediments of the Kunming Lake and the Aohai Lake, and pH, OM, AN, and TN influenced the archaeal distribution patterns considerably. *Bathyarchaeota* (MCG), *Euryarchaeota* and *Thaumarchaeota* were the most dominant phyla in the sediments of Kunming Lake and Aohai Lake and might be the key archaeal taxa, but their proportions differed among the sampling points. However, further research is needed to understand the ecological effects of the *Thaumarchaeota_Group C3* and Miscellaneous *Euryarchaeotic Group* (MEG) in lake sediments, as well as the different effects of hydrological characteristics and local geochemical characteristics on these sediment microbial communities. It is certain that the development of various research methods (such as macro genomics and proteomics) and the joint efforts of many interdisciplinary scientists will help elucidate biogeographic patterns and their ecological drivers of archaeal communities and populations in urban water bodies.
